# Serotype III *Streptococcus agalactiae* from Bovine Milk and Human Neonatal Infections^1^

**DOI:** 10.3201/eid1008.030917

**Published:** 2004-08

**Authors:** John F. Bohnsack, April A. Whiting, Gabriela Martinez, Nicola Jones, Elisabeth E. Adderson, Shauna Detrick, Anne J. Blaschke-Bonkowsky, Naiel Bisharat, Marcelo Gottschalk

**Affiliations:** * University of Utah Health Sciences Center, Salt Lake City, Utah, USA;; † Faculté de Médicine Vétérinaire, Université de Montréal, Saint-Hyacinthe, Québec, Canada;; ‡ John Radcliffe Hospital, Oxford, United Kingdom;; § St. Jude Children's Research Hospital, Memphis, Tennessee, USA; 1 Presented in part at the XVth Lancefield International Symposium on Streptococci and Streptococcal Diseases, October 6–11, 2002, Goa, India.

**Keywords:** Streptococcus agalactiae, group B streptococcus, bovine mastitis, phylogeny, *infB*, neonatal sepsis

## Abstract

*Streptococcus agalactiae* (group B streptococcus [GBS]) causes invasive human infections and bovine mastitis. This study examined the genetic relationship between bovine and human serotype III GBS by using molecular techniques that classify human serotype III GBS into four distinct phylogenetic lineages. Bovine serotype III GBS were largely contained in two lineages, which are distinct from the two major lineages (restriction digest types III-2 and III-3) that infect human neonates. One of the bovine lineages closely resembles the human III-1 lineage, whose members occasionally cause human neonatal infections. The bovine strains in the other lineage characteristically have an initiation factor IF2 gene (*infB*) H allele and multilocus sequence types that are not found in human GBS strains. Evidence suggests that this “H allele” lineage is related to the human III-3 lineage. These results support the assertion that human and bovine GBS are largely unrelated and provide further insight into the genetic relation between human and bovine GBS.

## Abstract 2

Although largely unrelated, many bovine type III GBS appear to share a common ancestor with an important human clone.

*Streptococcus agalactiae* (group B streptococcus [GBS]) is the major etiologic agent of invasive neonatal infections in humans in industrialized countries, causing sepsis, pneumonia, meningitis, osteomyelitis, and soft tissue infections (*1*). GBS has also been increasingly recognized as an important pathogen in immunocompromised and elderly persons (*2**,**3*). GBS emerged as an important cause of neonatal infections in the 1960s; before this time, it was mainly recognized as a cause of bovine mastitis (*4*). Most data suggest that GBS strains that infect humans are distinct from strains isolated from bovine sources, since bovine strains frequently cannot be typed with antisera to determine capsular polysaccharide serotype, often express protein antigens not found on human isolates, and tend to have different biochemical properties (*5**–**8*). The possibility remains, however, that subgroups of GBS infect both humans and cows. If so, these two closely associated hosts could act as reservoirs for each other and sites for the emergence of novel pathogens.

A number of molecular methods, including multilocus enzyme electrophoresis, pulsed-field gel electrophoresis (PFGE) of restriction enzyme digest products of genomic DNA, randomly amplified polymorphic DNA (RAPD) analysis, and multilocus sequence typing (MLST), have been used to demonstrate that the population of GBS that infects humans is highly clonal and limited to a relatively small number of phylogenetic lineages (*9**–**13*). Martinez and colleagues reported in 2000 that a large sample of GBS isolated from cows in Quebec Province in Canada could be classified into five major RAPD groups, which indicates that this sample of bovine GBS also comprised a limited number of lineages (*7*).

The Quebec sample is particularly useful for further investigating the relationship between bovine and human GBS because most typeable bovine isolates in the sample were serotype III. Serotype III GBS strains account for a substantial proportion of early-onset neonatal human GBS infections and almost all late-onset neonatal infections (*2*). Four distinct phylogenetic lineages of human serotype III GBS have been identified by PFGE of restriction digest patterns and designated restriction digest patterns type III-1, III-2, III-3, and III-4 (*11*). Human GBS strains can also be assigned to each restriction digest pattern type by a distinct set of molecular markers, which include analysis of nucleotide substitutions in the centrally conserved region of the translation *infB*, the presence or absence of the inserted sequences GBSi1 and IS*1548* in three chromosomal loci, and MLST (*9**,**11*).

RAPD analysis of human GBS isolates collected with the Quebec bovine isolates suggested that the serotype III bovine strains and human GBS strains were largely unrelated, although a definite conclusion was hindered by the small number of human isolates in the study. The obstacle presented by the small human sample size can now be circumvented by the use of the molecular markers described above that identify human phylogenetic lineages of GBS, but which have not yet been applied to the study of GBS from nonhuman sources. We reexamined the Quebec sample with these molecular markers to better understand the genetic relationship between bovine serotype III GBS and human serotype III GBS.

## Methods

### Bacterial Isolates

The serotype III GBS were isolated from bovine milk or from vaginal and rectal swab specimens from asymptomatic pregnant women in Quebec Province, Canada, during 1996 and 1997, as previously described (*7*). RAPD analysis of the 224 bovine GBS isolates had assigned 210 of the isolates to four RAPD groups (I–IV); the remaining 14 isolates were ungrouped (Figure). A total of 70 of the 82 original serotype III GBS strains were recovered for this study. The remaining GBS in this collection were not serotype III or were nontypeable. Bovine isolates were studied from all RAPD groups except RAPD group I, which contained a single bovine serotype III GBS isolate that could not be recovered. Genomic DNA was extracted with the Qiagen DNeasy Tissue Kit (Qiagen, Valencia, CA) from individual colonies grown overnight in broth.

**Figure:**
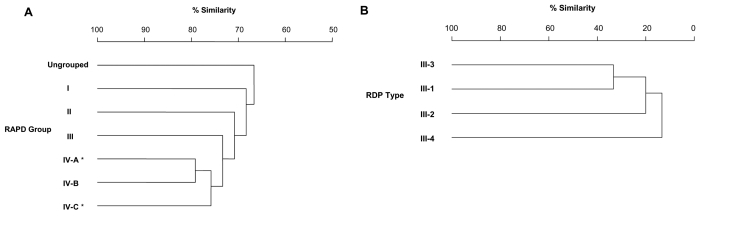
Simplified dendrograms illustrating the genetic relationship between human and bovine group B streptococcus. A) Dendrogram of Quebec sample derived by randomly amplified polymorphic DNA (RAPD) analysis; adapted from (7). All RAPD groups contain bovine serotype III GBS, but those marked by an asterisk also contain human isolates (see Table 2). B) Dendrogram of human restriction digest pattern types (RDP) of serotype III GBS derived by analysis of RDP of genomic DNA created by digestion with restriction enzyme *Sse*83871; adapted from (11).

### Molecular Analyses

The central portion of *infB* was amplified from bacterial DNA by polymerase chain reaction (PCR) with oligonucleotide primers, as described by Hedegaard et al. (*14*). Each amplicon was purified and sequenced.

The presence or absence of the inserted sequences GBSi1 and IS*1548* in the respective chromosomal loci was determined by using PCR of bacterial DNA with primer pairs flanking each of three sites, as previously described (*11*). In human serotype III GBS, the first locus is an internal region of *hylB*, the gene encoding hyaluronidase, which is either interrupted by IS*1548* or is intact. The second locus is the region between *scpB* and *lmb*, the genes encoding streptococcal C5a-ase and a laminin-binding protein, which either contains GBSi1, IS1548, or no insert. The third locus lies between *ftsY* and *sag0728*, two open reading frames (ORFs) with gene products of unknown function, which contains GBSi1 or no insert. The latter locus is either referred to as the AW-10 locus (as we refer to it) or the Y locus, according to Luan et al. (*15*).

MLST was carried out on 46 selected strains as previously described, and a sequence type was assigned to each strain (*9*). The sequences of the alleles that make up the novel MLST sequence types described in this manuscript can be found at http://sagalactiae.mlst.net (*9*). DNA dot blots were performed by using a 96-chamber vacuum manifold and [^32^P]-dCTP labeled probes as previously described (*16*).

The complete sequences of the *infB* gene H allele and the novel IS*1563*-like insertion sequence described in this article are available in the GenBank database under accession nos. AY429663 and AY437915, respectively.

## Results

### Analysis of *infB* Alleles in Bovine GBS

All human serotype III GBS have either the A or C allele of the gene encoding translation-initiation factor IF2 (*infB* allele, Table 1). Twenty-five of the 62 Quebec bovine isolates carried the A allele, and 2 carried the C allele, which raises the possibility that these 27 bovine GBS could be closely related to human GBS (Table 2). No strains bearing the B allele were identified, and only one strain carried the D allele. Serotype III GBS that bear the D allele are unlikely to be isolated from humans because all human GBS strains bearing the D allele have thus far been serotype Ia. The remaining 34 strains contained a previously unidentified *infB* allele, which we have designated the H allele. The *infB* H allele differs from other previously identified *infB* alleles (designated A–G) by two, three, or five nucleotide substitutions within the central conserved portion of the *infB* gene and most closely resembles the *infB* A allele (*11**,**14*). All 34 of the strains bearing the H alleles are bovine in origin (Table 2). The *infB* H allele has not been previously encountered in approximately 700 GBS isolates from human sources studied in our laboratory, including over 150 serotype III strains (unpub. data [11]; ). These observations suggest that GBS bearing the *infB* H allele rarely, if ever, colonize or infect humans.

**Table 1:** Characteristics of human serotype III GBS lineages^a^

RDP type^c^	*infB* allele^d^	Inserted sequence sites^b^			
AW-10	*hylB*	*scpb-lmb*	ST^e^
III-1	A	No insert	No insert	No insert	23, 25
III-2	A	No insert	IS*1548*	IS*1548*	19, 21
III-3	C	GBSi1	No insert	GBSi1	17, 29
III-4	A	No insert	No insert	GBSi1	1

^a^GBS, group B streptococcus. ^b^The presence of inserted sequences is determined at the three chromosomal locations (see Methods). ^c^RDP type refers to phylogenetic lineages originally defined by analysis of restriction digest patterns of chromosomal DNA. ^d^*infB* is the highly conserved gene encoding initiation-translation factor IF2. ^e^ST refers to the isolates' sequence type, based on the alleles of seven housekeeping genes identified by multilocus sequence typing.

**Table 2:** Distribution of *infB* alleles in serotype III GBS from different RAPD groups^a^

RAPD group^b^	Species of origin	Isolates studied	*infB* allele				
			A	B	C	D	H
II	Bovine	31	1	0	0	0	30
III	Bovine	4	3	0	0	1	0
IV-A	Bovine	19	19	0	0	0	0
	Human	1	1	0	0	0	0
IV-B	Bovine	2	0	0	0	0	2
IV-C	Bovine	4	2	0	2	0	0
	Human	7	5	0	2	0	0
Ungrouped	Bovine	2	0	0	0	0	2
	Total	70	31	0	4	1	34

^a^GBS, group B streptococcus. ^b^RAPD group identified by randomly amplified polymorphic DNA analysis.

### Relationship between *infB* Allele and RAPD Group

The possibility that the A or C allele bovine strains could be related to human GBS was investigated by examining the distribution of the bovine A or C allele isolates among the groups previously determined by RAPD analysis (Figure) (*7*). Of the 25 bovine A allele isolates, 19 were found in RAPD group IV-A, and 2 were found in RAPD group IV-C, which suggests that at least some of the bovine A allele isolates could be genetically related to human GBS since human A allele isolates are also found in these two RAPD clusters (Table 2, Figure). The two bovine C allele isolates are in RAPD group IV-C clustered with two human C allele isolates and thus appear closely related to human C allele isolates based on RAPD analysis. As expected, neither the single bovine D allele isolate nor the 34 bovine H allele isolates cluster with human isolates by RAPD analysis (Table 2).

### Identification of Bovine Serotype III GBS Related to Type III-1 GBS

The bovine A and C allele isolates were further examined by using techniques that distinguish the four known human serotype III GBS lineages. These techniques include determining the presence or absence of inserted sequences in three previously identified loci and determining the strains' sequence types by MLST (Table 1). Twenty-three of the 25 A allele bovine isolates appear closely related to human restriction digest pattern type III-1 strains because the isolates lack inserted sequences at any of the three loci examined and had a sequence type (ST) identical to those of previously studied III-1 strains (ST-23) or that was different by only one allele (ST-90, ST-92, or ST-94) (Table 3). Thus, a substantial proportion of the serotype III bovine GBS in this sample appear to come from a lineage that is associated with invasive neonatal disease, albeit rarely (*9**,**17**,**18*). Bovine III-1 strains appear to be genetically heterogeneous since they are found in RAPD groups III, IV-A, and IV-C, but no human III-1 strains were found in this sample; thus, the bovine III-1 strains most closely related to human III-1 strains could not be identified in this sample. Nineteen of the III-1 bovine isolates appear to lack the *scpb-lmb* locus because PCR with primers flanking the intergenic locus of *lmb* and *scpB* did not produce an amplicon.

**Table 3:** Inserted sequences and sequence type (ST) of bovine strains containing the *infB* A allele^a^

RAPD group	Isolate	Origin	Inserted sequence site				
			AW-10	*hylB*	*scpb-lmb*	ST	RDP type
II	SH-96-3696	Bovine	3,400^b^	None	None	67	Unknown^c^
III	NI-96-2836	Bovine	None	IS*1548*	IS*1548*	19	III-2-like
	SH-96-4807	Bovine	None	None	None	17	III-3-like
	RF-96-2997	Bovine	None	None	None	94	III-1
IV-A	1003A	Human	None	IS*1548*	IS*1548*	19	III-2
	AL-97-0498 SH-96-3417	Bovine	None	None	None	23	III-1
	All 17 others	Bovine	None	None	No product^d^	23,90,92	III-1-like
IV-C	NI-96-3213	Bovine	None	None	No product^d^	23	III-1-like
	ASS-96-666	Bovine	None	None	No product^d^	23	III-1-like
	1007B	Human	None	IS*1548*	IS*1548*/IS*1381*^e^	86	III-2
	1009A, 15888	Human	None	IS*1548*	IS*1548*	19	III-2
	13228, 1009B	Human	None	IS*1548* (truncated)^f^	IS*1548*	19	III-2

^a^RAPD, randomly amplified polymorphic DNA; RDP, restriction digest pattern. ^b^A 3,400-bp insert is also found in other strains from randomly amplified polymorphic DNA (RAPD) group II (see text and Table 5). ^c^This strain appears to be related to the H allele strains described in Table 5. ^d^No polymerase chain reaction product was produced from these strains in multiple attempts. ^e^In this strain, the insertion sequence IS*1381* is upstream from IS*1548* in the *scpb-lmb* locus. ^f^S*1548* in the *hylB* locus is missing 657 bp from the 3´ end of its open reading frame in this strain.

Seven of the A allele strains appear to be restriction digest pattern type III-2 strains on the basis of an analysis of inserted sequences and MLST (Table 3). Only one of these seven isolates, NI-96-2836, is of bovine origin, however, and is found in RAPD group III, whereas the six human A allele isolates are found in RAPD groups IV-A and IV-C. These data suggest that the bovine NI-96-2836 strain is genetically divergent from human III-2 strains, despite sharing the same *infB* allele, inserted sequences, and ST with human III-2 strains. These data also suggest that restriction digest pattern type III-2 strains, a major cause of human neonatal infections, are rarely, if ever, isolated from bovine milk.

One of the two remaining A allele strains, SH-96-4807, appears indistinguishable from III-3 strains on the basis of MLST because it has ST-17, which is characteristic of III-3 strains. Unlike III-3 strains, however, SH-96-4807 has no inserted sequences in any of the three sites and contains an *infB* A allele instead of the C allele typical of III-3 strains. The remaining A allele isolate, SH-96-3696, appears to be most closely related to H allele strains, on the basis of its RAPD group, inserted sequences, and ST. No bovine isolate related to restriction digest pattern type III-4 was found in this sample.

The four C allele isolates have the typical inserted sequences and ST (ST-17) found in restriction digest pattern type III-3 strains, with the exception of human isolate 1004A, which has a truncated form of IS*1548* in the *scpb-lmb* intergenic region (Table 4). These strains, which cluster together in RAPD Group IV-C, were isolated from both human and bovine sources. These data indicate that strains from the restriction digest pattern type III-3 lineage infect both humans and bovine udders but that bovine III-3 strains are rare. The only D allele strain has ST-93, an ST which differs by at least three alleles from all the STs previously described for human GBS, which again indicates that D allele serotype III GBS rarely or never colonize humans.

**Table 4:** Analysis of inserted sequences of strains containing the *infB* C allele^a^

RAPD	Isolate	Inserted sequence site					
		Origin	AW-10	*hylB*	*scpb-lmb*	ST	RDP type
IV-C	SF 96-5547	Bovine	GBSi1	700	GBSi1	17	III-3
	1004A	Human	GBSi1	700	IS*1548*^b^	17	III-3
	1000B	Human	GBSi1	700	GBSi1	17	III-3
	SF-96-4054	Bovine	GBSi1	700	GBSi1	17	III-3

^a^RAPD, randomly amplified polymorphic DNA; ST, sequence type; RDP, restriction digest pattern. ^b^An insert highly homologous to IS*1548* but lacking bp 603–1,301 was found in this strain. IS*1548* has not previously been found in this locus in III-3 strains.

### Analysis of H Allele Isolates

Ten H allele isolates were selected to represent strains from RAPD Group II, Group III, and the ungrouped isolates and analyzed in the same fashion. As shown in Table 5, all of the isolates studied have large inserts in the AW-10 site. The 1,700-bp inserts would be the correct size for the GBSi1 insert found in III-3 strains, and sequencing demonstrated an intact copy of GBSi1 in a 1,700-bp amplicon from one of the strains. A 3,000-bp insert from one strain was amplified and sequenced and found to comprise GBSi1, interrupted by an IS*3*-like insertion sequence identical to sequences found in both the genome M1 strain of *S. pyogenes* and the genome serotype V strain of *S. agalactiae* (*19**,**20*).

**Table 5:** Inserted sequences and sequence types of *infB* H allele strains^a^

RAPD	Isolate	Size of PCR product from inserted sequence sites (bp)			
		AW-10	*hylB*	*scpb-lmb*	ST
II	RF-96-2834	3,000^b^	700^c^	650^d^	61
	ASS-97-0701	3,000	700	650	61
	AL-96-1653	1,700^e^	700	650	61
	SF-96-6312	3,400^f^	700	650	61^g^
	SF-96-4396	1,700	700	650	91
	NI-96-2521	3,000	700	650	105
IV-B	ASS-96-659	3,000	700	No product	61^g^
	SH-96-5461	1,700	700	No product	61
Ungrouped	AL-96-2049	3,000	700	650	61
	NI-96-3329	3,000	700	650	61

^a^RAPD, randomly amplified polymorphic DNA; ST, sequence type. ^b^A 3,000-bp amplicon was sequenced and found to contain an IS*3*-like insertion sequence interrupting GBSi1. ^c^A 700-bp product from the *hylB* site indicates that there is no inserted sequence in the site. ^d^A 650-bp product from the *scpb-lmb* site indicates that there is no inserted sequence in the site. ^e^A 1,700-bp product was amplified and found to contain an intact copy of GBSi1. ^f^A 3,400-bp amplicon was sequenced and found to contain GBSi1 and an IS*1563*-like insertion sequence. ^g^The *glcK* (glucose kinase) gene in these two strains contains a mobile genetic element in the portion of *glcK* used to determine the ST of GBS strains. These two strains were found to be sequence type 61 (ST-61) after the inserted sequence was removed. The identical inserted sequence in the *glcK* gene has been found in other ST-61 bovine strains and is described elsewhere (21).

A 3,400-bp insert was sequenced and found to consist of an inserted sequence that has identical direct and inverted repeat sequences to the insertion sequence IS*1563*, but it has a predicted amino acid sequence that is 75% identical to that of IS*1563*. This IS*1563*-like insertion sequence is located upstream of GBSi1 exactly as IS*1563* is found upstream of GBSi1 in this locus in restriction digest pattern type II-2 strains (*11*).

The AW-10, *hylB*, and *scpb-lmb* loci were studied in the remaining H allele strains not shown in Table 5. No inserted sequences were found in the AW-10 site in seven isolates, while the remaining strains have either the 1,700-bp, 3,000-bp, or 3,400-bp inserts at AW-10. No inserted sequences were found in the *hylB* and *scpb-lmb* sites in any of the H allele strains. Two of the H allele strains, both of which are in RAPD group IV-B, appear to lack the *scpb-lmb* locus.

MLST shows that 8 of these 10 H allele isolates are ST-61. ST-61 isolates are found in RAPD groups II, IV, and in ungrouped isolates, which indicates genetic divergence among H allele strains that is detected by RAPD but not by MLST. ST-61 differs from ST-67 by one allele, which confirms that SH-96-3696, the A allele strain found in RAPD II that has ST-67, is in the same clonal complex as the H allele serotype III strains. SH-96-3696 also has a 3,400-bp insert at AW-10, and no insert at the other two sites, which provides further evidence that this isolate is related to H allele strains. The other two STs found in the H allele strains, ST-91 and ST-105, differ from ST-61 by two alleles. Thus, the H allele strains are clonally related.

### III-3–specific Sequence Tags in H Allele Serotype III GBS

ST-61, the most common ST of the H allele strains, differs by two alleles from ST-17, the most common ST of III-3 strains. This two-allele difference in ST and the observation that isolates from these two lineages both have GBSi1 in the AW-10 site led us to search for other evidence that the two lineages are related.

We previously described 10 short sequence tags that are found in all III-3 strains but not in III-1 or III-2 strains (*16*). We therefore performed dot-blot hybridization with eight of these probes on a selection of A, H, and C allele strains from this sample to determine the distribution of the III-3–specific sequences among these various lineages. As expected, all of the III-3–specific probes hybridized with every C allele strain tested, whereas the III-3 probes hybridized rarely or never with the A allele strains. In contrast, seven of the eight III-3 probes hybridized with almost all the H allele strains (Table 6).

**Table 6:** Distribution of III-3–specific sequence tags

III-3–specific sequence	*infB* allele^a^		
	H	A	C
DY-1	10/10	0/10	3/3
DY-3	2/10	1/10	3/3
DY-11	7/10	0/10	3/3
AA 3.8	10/10	2/10	3/3
AA 3.14	10/10	0/10	3/3
AA 3.16	9/10	1/10	3/3
AA 4.1	10/10	2/10	3/3
AA 4.13	10/10	0/10	3/3

^a^Number of strains with sequence tag by DNA hybridization/number of strains tested.

## Discussion

A key finding of this investigation is that the serotype III GBS strains isolated from bovine milk in this sample are largely genetically distinct from the serotype III GBS strains that commonly infect humans. The two most common lineages of serotype III GBS that colonize women and infect infants in the United States and Japan are restriction digest pattern types III-2 and III-3, whereas III-1 and III-4 strains are rarely isolated from the genitourinary tract of women and are rarely associated with invasive disease (*11**,**16*).

Only one bovine strain that resembled RDP type III-2 GBS was identified in the 67 isolates, and RAPD analysis indicates that this isolate is distinct from the 7 human III-2 strains in the sample. Thus, III-2 strains that infect humans are unlikely to infect bovine udders. Only two bovine strains were identified that had the genotypic characteristics of restriction digest pattern type III-3 strains. These two strains appear closely related to two human III-3 isolates by RAPD analysis, which makes it likely that III-3 strains can infect bovine udders, but they appear to do so infrequently.

We also found that the vast majority of bovine serotype III GBS in this sample belong to one of two major phylogenetic lineages. A separate study of bovine GBS isolates collected in the United Kingdom used MLST to identify two clonal complexes that are similar, if not identical, to the two lineages identified here; this finding suggests that two clones of GBS may predominate among bovine mastitis caused by GBS in North America and England (*21*).

The data presented here show that the first bovine lineage is closely related to restriction digest pattern type III-1 GBS (Table 7). The lack of human III-1 strains in this sample makes it difficult to use the RAPD analysis to determine how closely these III-1–like bovine strains resemble human III-1 strains. That no III-1 human isolates were found in this small sample is not surprising because human III-1 isolates rarely colonize the female genitourinary tract (although they occasionally cause neonatal infections) (*9**,**17**,**18*). Most III-1 bovine isolates in the sample studied here appear to lack the *scpb-lmb* locus, a finding consistent with that reported by Francken et al., who showed that absence of a putative composite transposon that contains the *scpB* and *lmb* genes is a feature of bovine GBS (*22*). Possibly only a few of the III-1 bovine GBS are capable of infecting both humans and cows, since all human GBS appear to have the *scpb-lmb* locus.

**Table 7:** Similarities and differences between the two major bovine lineages and related human lineages

Bovine lineage	Human lineage	Similarities	Differences
III-1	III-1	Same *infB* allele (A) No inserted sequences in AW-10, *hylb* or *scpb-lmb* sites Sequence types (STs) the same or differ by <1 allele by multilocus sequence typing (MLST)	Many bovine III-1 strains lack *scpb-lmb*
H allele	III-3	GBSi1 inserted in AW-10 site No inserted sequence in *hylb* STs differ by only two alleles by MLST III-3 specific sequence tags in genome	Different *infB* allele (H vs C) BSi1 not found in *scpb-lmb* Additional inserted sequences or none in AW-10 site with GBSi1 Some bovine H allele strains lack *scp-lmb*

The other major bovine serotype III GBS lineage in this sample is composed of strains that possess an *infB* H allele, with the exception of a single A allele strain that, on the basis of RAPD and MLST, appears closely related to the H allele strains. The H allele strains have STs that differ from each other by no more than two alleles, which indicates that these strains are likely to have a recent common ancestor.

We believe that strains in this lineage are unlikely to colonize or infect humans since we have not identified an *infB* H allele strain in >160 human serotype III GBS isolates, and since the STs of the bacteria in this lineage were not found in a large sample of human GBS obtained from diverse geographic areas (*9*). However, the major ST (ST-61) found in this lineage differs from the major ST of III-3 strains by only two alleles, which suggests that strains from this group may share a relatively recent common ancestor. H allele strains were found to contain previously identified III-3–specific sequence tags, which supports this hypothesis.

The identification of two III-3 strains of bovine origin and a third bovine strain (SH-96-4807) that is genetically distinct from III-3 strains but with the ST typical of III-3 strains (ST-17) supports the concept that III-3 strains share a recent common ancestor with H allele strains and retain some genetic traits necessary for bovine udder colonization. No bovine III-2 strains were identified, but a single bovine strain (NI-96-2836) appears to be related to strains in the III-2 lineage by all molecular markers (although RAPD analysis found it to be clearly distinct from human III-2 isolates). These III-3–like and III-2–like bovine strains are both in RAPD group III (Table 3) and thus appear by RAPD analysis to be more related to each other than to other major bovine lineages or to human isolates. RAPD analysis also suggests that human III-3 strains are more related to human III-2 strains than to the H allele strains, despite the observation that MLST puts human III-3 strains at a greater phylogenetic distance from III-2 strains than from the H allele strains.

The clustering together of human III-2 and III-3 isolates by RAPD analysis, despite their clear distinction by MLST, leads us to hypothesize that common genetic determinants that account for host tropism (human versus bovine) have been acquired by both the III-2 and III-3 lineages and strongly influence the clustering of isolates, as shown by RAPD analysis. If so, the two bovine isolates that closely resemble III-3 and III-2 strains by MLST, but which resemble each other more closely by RAPD analysis, may represent intermediate genotypes between GBS lineages that have a more clear-cut tropism for either humans or bovines. The exact relationship between these human and bovine lineages, and genes important for host tropism, could be clarified by comparative genomics. Such studies, along with further studies of bovine GBS lineages of other serotypes, could also provide insight into the exact relationship between human and bovine strains and help determine whether these hosts act as reservoirs for each other's pathogenetic lineages and for the emergence of new pathogenic clones.

This study was supported by the following grants: USPHS NIH RO1 AI 40918 (J.F.B., E.E.A.); Thrasher Research Fund (J.F.B.); Cancer Center Support CORE Grant P30 CA 21765 (E.E.A.); and the American Lebanese Syrian Associated Charities (E.E.A.).
